# We Do Not Eat Alone: Formation and Maturation of the Oral Microbiota

**DOI:** 10.3390/biology9010017

**Published:** 2020-01-13

**Authors:** Luca Fiorillo

**Affiliations:** Department of Biomedical and Dental Sciences, Morphological and Functional Images, University of Messina, Policlinico G. Martino, Via Consolare Valeria, 98100 Messina, Italy; lfiorillo@unime.it

**Keywords:** oral hygiene, oral cavity, oral surgery, oral health, oral medicine, biofilm, microbiota, dental plaque, tooth socket, dental calculus

## Abstract

From the earliest moments of life, contact with the outside world and with other individuals invalidates the sterility of the oral cavity. The oral cavity passes from a sterility condition, that is present only during intrauterine life, to a condition in which a microbiota organizes and evolves itself, accompanying the person throughout their life. Depending on a patient’s age, systemic conditions and/or oral conditions, different characteristics of the oral microbiome are shown. By verifying and analyzing this process it is possible to understand what is at the basis of the etiopathogenesis of some oral pathologies, and also the function of the oral microbiome.

The anatomical diversity of the oral cavity forms different habitats, with different physical and chemical factors. These niches (lips, cheeks, palate, teeth, gingival sulcus) are suitable for different microbial populations. It is necessary to consider that the oral microbiota has great plasticity over time; both short term (during a day), and long term (during the lifetime). Analyzing the oral microbiota, and the different habitats, it can be easily concluded that the colonizable surfaces are different [1,2,3,4].

In fact, the oral cavity is characterized by hard tissue, such as enamel, which does not exfoliate, and thus allows for a stable colonization, and soft tissues, which undergo renewal and exfoliation and a final habitat, defined as the crevicular area, which has specific characteristics with respect to bacterial colonization.

The oral cavity is also characterized by a high humidity, guaranteed by the presence of saliva and crevicular liquid. Saliva has a cleansing and other important functions during the chewing phases. It is produced in a quantity of about 800–1500 mL/day. Although saliva has an important antibacterial function, it contains proteins or glycoproteins, which favor the formation of the acquired enamel film (enamel pellicle). The enamel film allows the adhesion of bacteria and therefore, the formation of plaque. On the other hand, the crevicular fluid is an exudate of plasmatic origin [5,6,7,8,9].

There are chemical and physical factors that influence the oral microbiota, such as temperature, pH, anaerobic conditions, age, hormonal variations, hygiene, type of diet, presence of systemic diseases or the use of drugs, all of which affect bacterial colonization (Figure 1) [9,10,11,12,13,14,15,16].

While the oral cavity is host to microbes that belong to different species, in general, the predominant bacteria in the mouth are Streptococci. The oral cavity is sterile at birth, but bacterial colonization already begins within 6–10 h, and undergoes maturation and changes over the following hours. The changes of the oral microbiota are not due only to the passage of time, but as previously specified, also due to other co-factors, and to the oral anatomical condition changes. The newborn’s edentulous mouth has no teeth, no hard surfaces, and anaerobic condition habitats, such as the gingival sulcus. For this reason, the anaerobic bacteria in these phases are scarce (Veillonella Fusobacteria and Peptostreptococci), to the detriment of the optional aerobic-anaerobic colonizers such as Streptococci, in particular the *Streptococcus salivarius*. During the eruption phases, the microbiome changes, as a result of the colonization of not exfoliating hard surfaces. This change could occur by *Streptococcus sanguis*, *Neisseria sicca*, *Streptococcus mitis*, or *Streptococcus mutans* that leads to the formation of the first plaque. Tartar originates from plaque calcification [17,18,19].

Subsequent to the formation of the enamel film, which takes place a few seconds after having performed the oral hygiene maneuvers, the tooth surfaces are colonized. The initial phase lasts about 8 h, in these phases the Streptococci (*S. sanguis*, *S. mitis*) and some Neisserie (*N. sicca*) lead to the formation of microcolonies. The subsequent phases see the maturation of the plaque, which leads to non-quantitative but qualitative changes. In fact, anaerobic conditions are realized, and receptor structures are formed which indicate a bacterial interaction. The formation of plaque layers makes the bacteria resistant to both drugs and host responses [20,21,22,23].

Following this, it is possible to distinguish a supragingival plaque from a subgingival plaque. The supragingival plaque is mainly composed of streptococci (*S. sanguis*, *S. mitis*, *S. mutans*); actinomycetes (*Actinomyces viscosus*) and Veilonelle. It is, however, necessary to consider that some of these bacterial species are antagonistic, and for this reason, they tend to colonize different habitats, for example, the *Streptococcus mutans* is present on the occlusal and approximal faces of the teeth, while the *Streptococcus sanguis* is on the smooth teeth surfaces. Subgingival plaque, characterized by conditions of anaerobiosis, is highly variable. The structure of the subgingival plaque has some similarities with that of the supragingival plaque, especially when it comes to plaque associated with gingivitis without the formation of a deep sulcus. The bacteria include Gram-positive and Gram-negative cocci and filamentous organisms. Spirochetes and various flagellated bacteria could be encountered, especially in the most apical areas of the plaque. The most superficial layer is often less densely colonized, and the leukocytes are regularly interposed between the plaque and the epithelial lining of the gingival sulcus. When a periodontal sulcus forms, the appearance of subgingival bacterial deposits becomes complex [24]. In this case, the tooth surface could be represented by both the enamel and the cement, from which the periodontal fibers have been detached. Filamentous microorganisms predominate in this layer of plaque, but cocci and rods are also found. On the other hand, in the deeper parts of the periodontal sulcus, the filamentous microorganisms become increasingly scarce, and in fact, seem to be absent in the most apical portion. Instead, the dense bacterial deposit that faces the tooth surface is dominated by smaller microorganisms, with no particular orientation. It is precisely during these phases of colonization that it is possible to create the conditions for the formation of colonies of aggressive bacteria against host tissues, such as *Campylobacter rectus*, *Prevotella Intermedia* and *Porphyromonas gingivalis* [25,26,27,28,29,30]. 

Bacterial plaque is not only formed on the surface of natural teeth, but also on artificial surfaces exposed to the oral environment, including implant surfaces. The similarities between peri-implant microbial and subgingival deposits have been clearly demonstrated by cross-sectional and longitudinal studies, and it is possible to state that the structure of peri-implant plaque deposits may resemble those encountered in the subgingival environment. The presence of a complex biofilm could have repercussions at the systemic level, or even have repercussions on surgical operations performed in the oral cavity [31,32,33,34,35,36,37,38,39].

In addition, bacterial metabolism of the multiple species found in plaque could lead to the formation of catabolites that could damage oral tissues [40,41,42,43,44].

Surely being able to fully understand these mechanisms could lead to therapies and preventive techniques aimed at the formation of a less aggressive plaque, or to inhibit these phenomena.

## Figures and Tables

**Figure 1:**
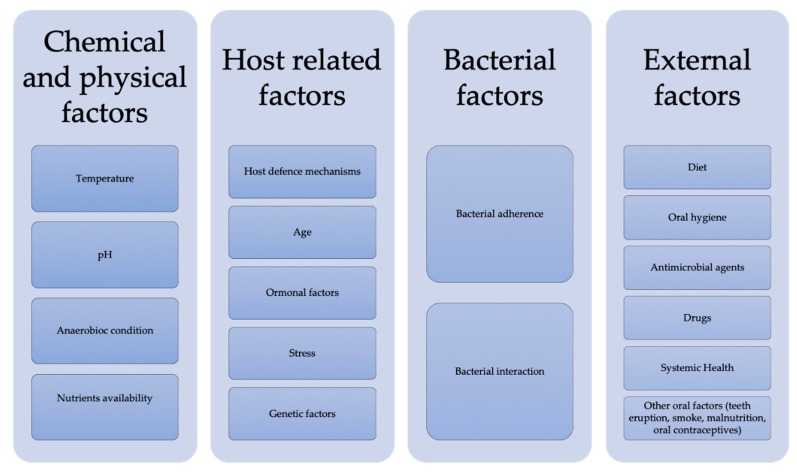
Factors that influence the oral ecosystem.
